# Quantifying bone marrow fat using standard T1-weighted magnetic resonance images in children with typical development and in children with cerebral palsy

**DOI:** 10.1038/s41598-019-57030-5

**Published:** 2020-03-09

**Authors:** Chuan Zhang, Jill M. Slade, Freeman Miller, Christopher M. Modlesky

**Affiliations:** 1 Department of Kinesiology, University of Georgia, Athens, GA USA; 2 Department of Radiology, Michigan State University, East Lansing, MI USA; 3 Department of Orthopedics, Nemours AI duPont Hospital for Children, Wilmington, DE USA

**Keywords:** Musculoskeletal system, Growth disorders, Paediatric research

## Abstract

Excess bone marrow adiposity may have a negative effect on bone growth and development. The aim of this study was to determine whether a procedure using standard T1-weighted magnetic resonance images provides an accurate estimate of bone marrow fat in children with typical development and in children with mild spastic cerebral palsy (CP; n = 15/group; 4–11 y). Magnetic resonance imaging was used to acquire T1-weighted images. It was also used to acquire fat and water images using an iterative decomposition of water and fat with echo asymmetry and least-squares estimation (IDEAL) technique. Bone marrow fat volume and fat fraction in the middle-third of the tibia were determined using the standard T1-weighted images (BMFV_T1_ and BMFF_T1_, respectively) and the fat and water images (BMFV_IDEAL_ and BMFF_IDEAL_, respectively). In both groups, BMFV_T1_ was highly correlated with (both *r* > 0.99, *p* < 0.001) and not different from (both *p* > 0.05) BMFV_IDEAL_. In both groups, BMFF_T1_ was moderately correlated with (both *r* = 0.71, *p* < 0.01) and not different from (both *p* > 0.05) BMFF_IDEAL_. There was no group difference in BMFV_T1_ or BMFV_IDEAL_ (both *p* > 0.05). BMFF_IDEAL_ was higher in children with CP (*p* < 0.05), but there was no group difference in BMFF_T1_ (*p* > 0.05). We conclude that a procedure using standard T1-weighted magnetic resonance images can produce estimates of bone marrow fat volume similar to estimates from the IDEAL technique in children. However, it is less sensitive to variation in the bone marrow fat fraction.

## Introduction

Bone marrow fat has drawn considerable attention in recent years, mainly due to its potential role in osteoporosis^1–3^. Bone marrow contains mesenchymal stem cells, which have the capacity to differentiate into osteoblasts or adipocytes. Adipocytes can form at the expense of osteoblasts^4^ when exposed to environmental factors associated with deficits in bone mass, strength and architecture, such as estrogen deficiency, glucocorticoids and mechanical unloading. A number of studies have shown that human bone marrow fat is negatively related to areal^5,6^ and volumetric bone mineral density (BMD)^7^ and is higher in those with osteoporosis than in those without osteoporosis^8^. It is likely that the environmental effect on bone marrow fat infiltration begins early in life. This notion is supported by the observation that children with movement disorders, such as cerebral palsy (CP), have a higher concentration of bone marrow fat^9^, as well as lower BMD^10^, lower bone strength^9,11,12^ and less developed bone architecture^11–14^ than typically developing children.

Fortunately, the development of new magnetic resonance imaging (MRI) techniques during the past twenty years, such as proton magnetic resonance spectroscopy (MRS) and iterative decomposition of water and fat with echo asymmetry and least-squares estimation (IDEAL)^15^, which are forms of chemical shift imaging, allow for the quantification of bone marrow fat in humans. Unfortunately, not all MRI scanners have the capability to perform these advanced imaging techniques. Instead, some studies have used standard T1-weighted magnetic resonance images, which are accessible on all MRI scanners, and applied a threshold based on fat signal intensity to assess bone marrow fat^16–18^. However, the use of standard T1-weighted images to assess bone marrow fat in children has not been validated. Therefore, in this study we sought to test the validity of using standard T1-weighted images to quantify bone marrow fat in children.

The purpose of this study was to determine whether a method using standard T1-weighted magnetic resonance images provides an accurate estimate of bone marrow fat in children who are typically developing, and in children with CP, a population that has been shown to have elevated bone marrow fat compared to their typically developing peers^9^.

## Methods

### Participants

Thirty children participated in the study. Fifteen were children with spastic CP who were able to ambulate independently or with an assistive device (n = 4 girls, n = 11 boys) and 15 were typically developing children without known neurological disorders and similar in age and sex to children with CP. This study was approved by the Institutional Review Board at the Nemours AI duPont Hospital for Children, Wilmington, DE. All methods were carried out in accordance with relevant guidelines and regulations. Informed consent was acquired from a parent or a legal guardian for study participation and assent was acquired from the participant, if >7 years of age and able, before any data collection was performed.

### Anthropometrics

Height and body mass were measured while participants were wearing minimal clothing. Height was assessed with a stadiometer (Seca 217; Seca GmbH & Co. KG., Hamburg, GER) to the nearest 0.1 cm. Body mass was assessed with a digital weight scale (Detecto 6550, Cardinal Scale, Webb City, MO) to the nearest 0.2 kg. Body mass index (BMI) was subsequently calculated based on the obtained height and body mass. Height, body mass and BMI percentiles were calculated for each participant based on the Centers for Disease Control and Prevention growth charts^19^.

### Sexual maturity

A physician assistant performed sexual maturity assessments using the Tanner staging technique^20^. Tanner staging is a scale ranging from 1 to 5 with higher numbers indicating more advanced sexual development. In boys, pubic hair and testicular/penile development were assessed. In girls, pubic hair and breast development were assessed.

### Gross motor function

The gross motor function classification system (GMFCS) was used to assess gross motor function of children with CP^21,22^. GMFCS is a scale ranging from I to V with higher numbers indicating more compromised motor function. Briefly, a child with the ability to walk without restrictions, but with limitations in more advanced motor skills, was classified as GMFCS I. A child with the ability to walk without assistive devices, but with limitations walking outdoors and in the community, was classified as GMFCS II. A child with the ability to walk with an assistive device, but with limitations walking outdoors and in the community, was classified as GMFCS III. All children with CP included in the current study were classified as GMFCS I-III.

### Magnetic resonance imaging

Magnetic resonance imaging (1.5 T; GE, Waukesha, WI) was used to assess bone marrow fat in the midtibia of the more affected limb in children with CP and in the non-dominant limb in controls. The BodyFIX (Medical Intelligence Inc, Schwabmunchen, Germany) was used to secure the children from the waist down to minimize the influence of movement, as previously described^23^. A three-plane localizer was first used to identify the region of interest. Axial images (0.5 cm thick with 0.5 cm spacing) were collected from the tibia plateau to the medial malleolar articular surface using a semiflex long bone array coil (ScanMed, Omaha, NE) and two different protocols. The first protocol (fast spin echo, TR = 650 ms, TE = 14 ms, FOV = 12 cm, NEX = 3, BW = 15.63 kHz, frequency = 512, phase = 256) yielded standard T1-weighted images. The second protocol, which used the IDEAL technique (fast-spin-echo, 2D, 3 echoes, TR = 600 ms, TE = min full, FOV = 12 cm, NEX = 2, BW = 31.25 kHz, echo time = 16.8 ms, frequency = 320, phase = 224), yielded fat and water images. The total number of images collected was based on tibia length and ranged from 24 to 34.

**Figure 1:**
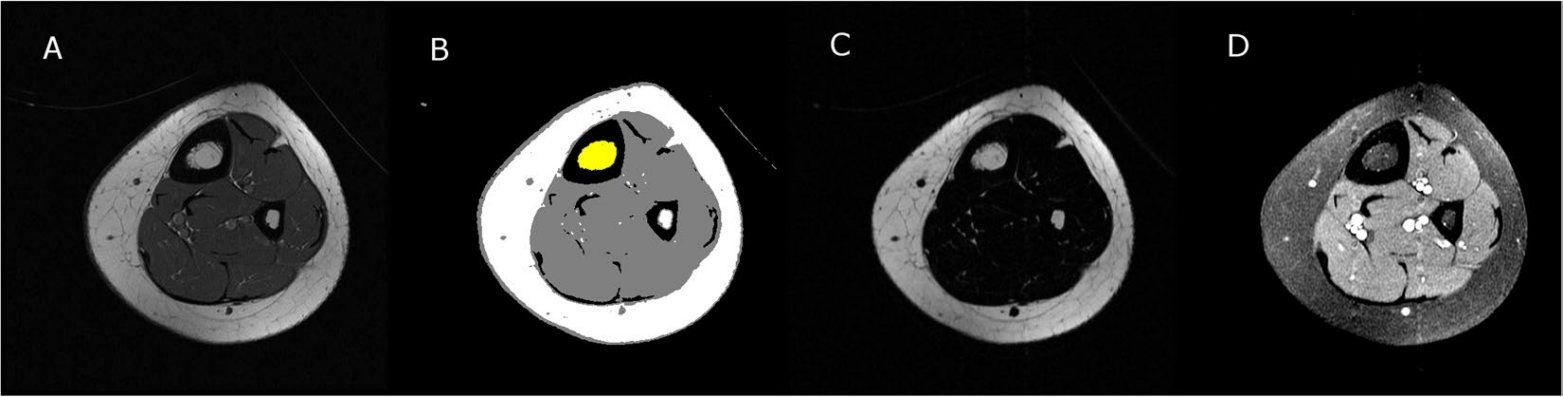
Visual description of the procedure to determine bone marrow fat fraction using fat and water images generated from the IDEAL technique and custom software. First, a raw T1-weighted magnetic resonance image (**A**) was filtered and then used to identify the bone marrow area (yellow region in **B**). The identified voxels were then applied to fat (**C**) and water (**D**) images to determine bone marrow fat area and to calculate bone marrow fat fraction. Bone marrow fat volume was quantified by accounting for the number of images, image thickness and spacing between images.

Total bone marrow volume was determined using the standard T1-weighted images and a custom program developed using Interactive Data Language (IDL; Research Systems, Inc., Boulder, CO). The procedure to determine bone marrow fat fraction (BMFF_IDEAL_) and bone fat marrow volume (BMFV_IDEAL_) using fat and water images generated from the IDEAL technique and the same custom program is briefly described in Fig. 1. The bone marrow was first identified using the T1-weighted images that were median filtered and separated from other tissues using a fuzzy-clustering algorithm. The T1 and IDEAL images were co-registered and the region (or mask) defined from the T1 images was applied to the corresponding fat and water images. The mean signal intensity (SI) was extracted from the fat and water images. BMFF_IDEAL_ of each slice was calculated using the following equation^24^:

1 $$BMF{F}_{IDEAL}=\frac{SI\,from\,fat\,images}{(SI\,from\,fat\,images+SI\,from\,water\,images)}\times 100$$

BMFF_IDEAL_ was multiplied by the bone marrow area identified from T1-weighted images to estimate bone marrow fat area. The bone marrow fat volume for the midtibia was then calculated.

**Figure 2:**
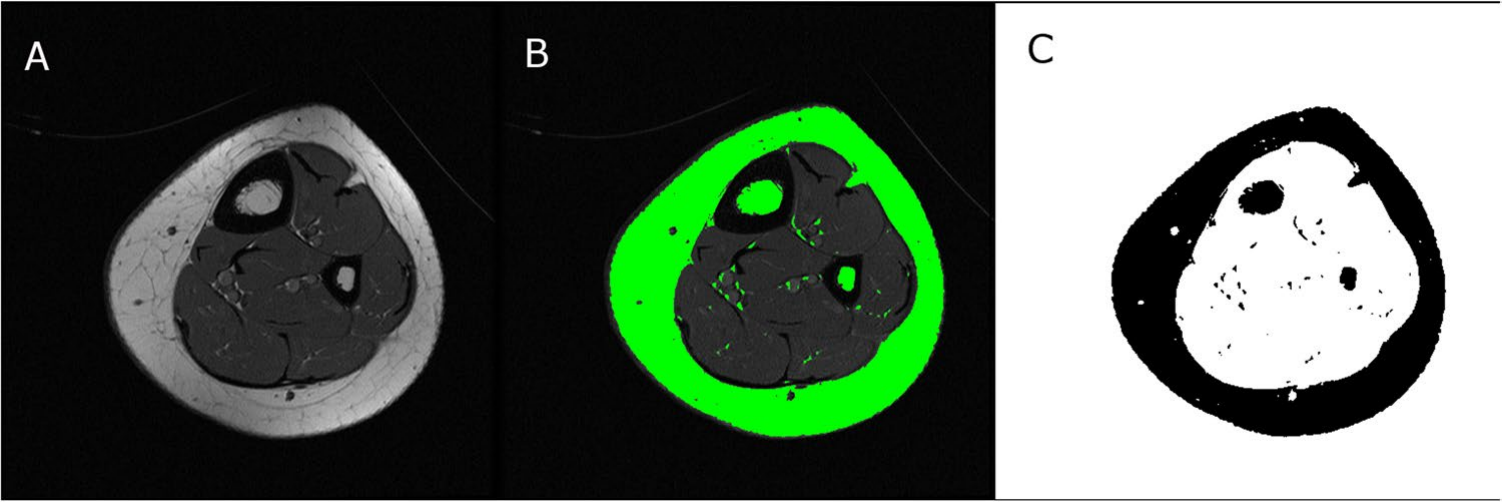
Visual description of the procedure to determine bone marrow fat fraction using standard T1-weighted magnetic resonance images and the ImageJ software. First, a raw image (**A**) was used to determine a segmentation threshold that best matched the subcutaneous fat area (green outer ring in **B**). The image was then binarized and bone marrow fat voxels identified at the same threshold as the subcutaneous fat area threshold were identified (**C**) and used to estimate bone marrow fat area. Bone marrow fat volume was quantified by accounting for the number of images, image thickness and spacing between images.

The procedure to determine bone marrow fat fraction (BMFF_T1_) and bone marrow fat volume (BMFV_T1_) using T1-weighted images and the open source software ImageJ^25^ is briefly described in Fig. 2. The raw images were first imported to ImageJ and then the threshold that best matched the segmentation of subcutaneous fat area was determined. The threshold was used to estimate bone marrow fat area from which BMFV_T1_ for the midtibia was calculated. BMFF_T1_ was calculated for each image by dividing bone marrow fat area by bone marrow area and multiplying by 100. The average BMFF_T1_ for all images evaluated is reported. The test-retest reliability for bone marrow fat fraction in the midtibia was previously determined in a sample of four children with CP and four typically developing children (5 to 11 years of age) tested twice, 6 months apart. The intraclass correlation was > 0.96 and the coefficient of variation was 0.04%^9^.

### Statistics

Data were analyzed using SPSS version 24.0 (IBM Corp, Armonk, NY). Physical characteristics data in typically developing children and in children with CP were checked for normality. If the data were normality distributed, group comparisons were made using independent t-tests. If data were not normally distributed, group comparisons were made using the Mann-Whitney test.

To determine the accuracy of using T1-weighted images to estimate bone marrow fat volume and concentration, data from each group were first compared separately. Differences between BMFV_T1_ and BMFV_IDEAL_ and between BMFF_T1_ and BMFF_IDEAL_ were determined using paired t-tests in each group. In addition, Pearson correlation analysis was performed to determine the association of bone marrow fat yielded from the two techniques. Moreover, for each group, bone marrow fat fraction and area estimated from T1-weighted magnetic resonance images were compared to estimates from fat and water images and the IDEAL technique using Bland-Altman plots^26,27^. Finally, between groups analyses for BMFF_T1,_ BMFF_IDEAL_, BMFV_T1_ and BMFV_IDEAL_ were also performed using independent t-tests. All data were presented as mean ± SD in tables. Data were reported as mean ± SE in figures. The magnitude of the effects was determined by Cohen’s d (*d*) whenever applicable, with 0.2, 0.5 and 0.8 representing small, moderate and large effect sizes, respectively^28^.

## Results

All physical characteristics are summarized in Table 1. No between group differences were detected between typically developing children and children with CP except for height percentile and body mass percentile, which were significantly lower in children with CP (both *p* < 0.05).

**Table 1:** Physical characteristics in children with cerebral palsy (CP) and in typically developing children (Con).

	CP (n = 15)	Con (n = 15)	*d*	*p*
Ages (years)	8.4 ± 2.4	8.2 ± 2.2	0.083	0.832
Tanner stage (I/II/III)				
Pubic hair	10/4/1	10/5		
Testicular-penile/breast	11/4	12/2/1		
Height (m)	1.22 ± 0.14	1.30 ± 0.11	0.635	0.137
Height (%)	18 ± 27	58 ± 29	1.428	**<0.001**
Body mass (kg)	26.5 ± 10.2	28.6 ± 7.0	0.240	0.524
Body mass (%)	35 ± 35	59 ± 25	0.789	**0.037**
BMI (kg/m²)	17.1 ± 3.4	16.8 ± 2.4	0.102	0.794
BMI (%)	53 ± 36	53 ± 29	0.004	0.991
GMFCS (I/II/III)	7/6/2	—		

BMI, body mass index; GMFCS, gross motor function classification system. % for height, body mass and BMI reflects the percentile relative to age- and sex- based norms. Significant differences are bolded.

**Figure 3:**
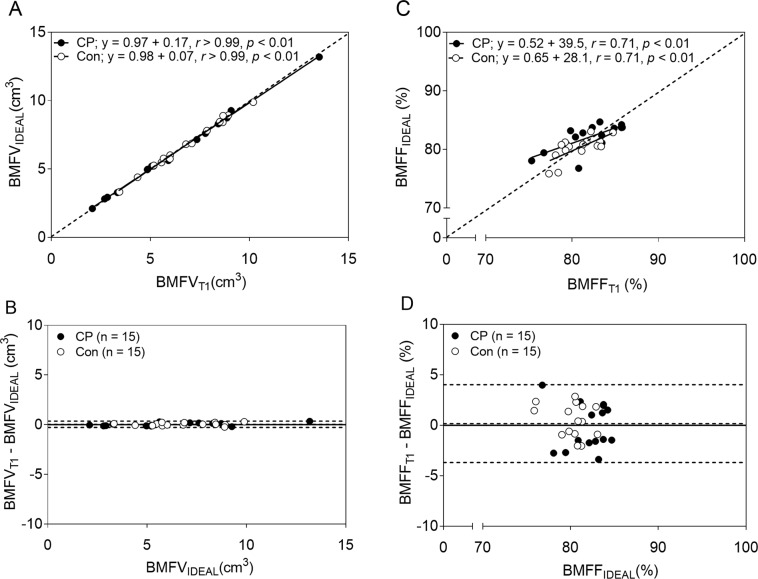
Comparison of bone marrow fat estimated by the procedure using standard T1-weighted magnetic resonance images and the procedure using fat and water images generated from the IDEAL technique. (**A**) The scatter plot shows strong agreement between bone marrow fat volume estimated from the standard T1-weighted images (BMFV_T1_) and bone marrow fat volume estimated using fat and water images from the IDEAL technique (BMFV_IDEAL_). (**B**) The Bland-Altman plot shows the level of agreement between BMFV_T1_ and BMFV_IDEAL_. (**C**) The scatter plot shows moderate agreement between bone marrow fat fraction estimated from the standard T1-weighted magnetic images (BMFF_T1_) and bone marrow fat volume estimated using fat and water images (BMFF_IDEAL_). (**D**) The Bland-Altman plot shows the level of agreement between BMFF_T1_ and BMFF_IDEAL_. The dotted lines in panels A and C represent the lines of identity. The dotted lines in panels B and D indicate the mean difference ± 2 SD between estimates from the standard T1-weighted and the fat and water images. The solid lines indicate no difference for estimation between the two methods.

There was no difference between BMFV_T1_ and BMFV_IDEAL_ in children with CP (*d* = 0.144, *p* = 0.978) or in typically developing children (*d* = 0.041, *p* > 0.99). The scatter plot in Fig. 3A shows that BMFV_T1_ was highly correlated with BMFV_IDEAL_ (*r* > 0.99, *p* < 0.001 for both groups). The Bland-Altman plot in Fig. 3B confirms the validity of BMFV_T1_ when compared to BMFV_IDEAL_ showing no bias between the two measures (mean difference = 0.04 cm^3^) and a very small degree of variability in the difference between them (SD of the difference = 0.16 cm^3^).

There was no significant difference between BMFF_T1_ and BMFF_IDEAL_ in children with CP (*d* = 0.055, *p* = 0.881) or in typically developing children (*d* = 0.234, *p* = 0.527). The scatter plot in Fig. 3C shows that BMFF_T1_ was moderately correlated with BMFV_IDEAL_ (*r* = 0.71, *p* = 0.003 in both groups). The Bland-Altman plot in Fig. 3D suggests that there was no bias between BMFF_T1_ and BMFF_IDEAL_ (mean difference = 0.17%); however, there was more variability between them (SD of the difference = 1.97%) than the variability shown between BMFV_T1_ and BMFV_IDEAL_.

**Figure 4:**
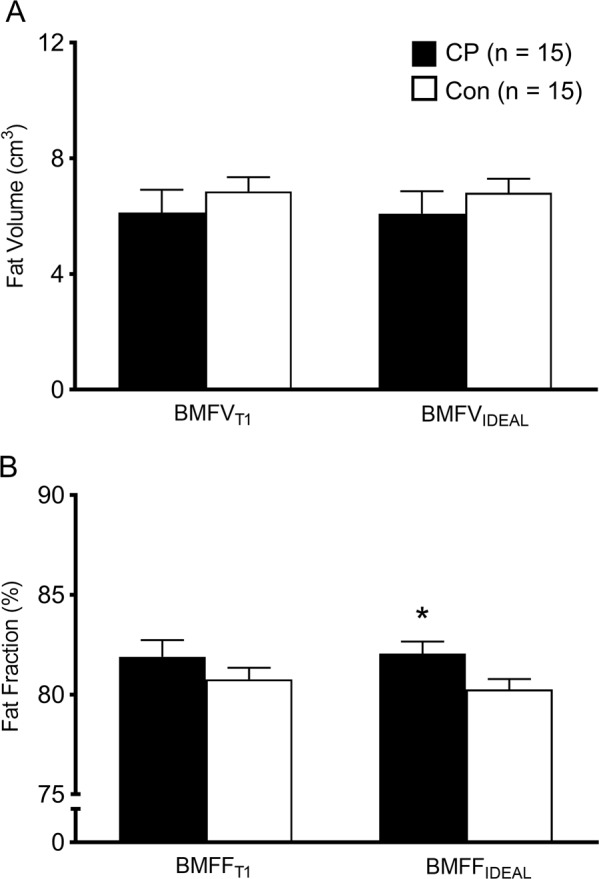
Bone marrow fat volume (**A**) and fat fraction (**B**) estimated by the procedure using standard T1-weighted images (BMFV_T1_ and BMFF_T1_, respectively) and by the procedure using fat and water images generated from the IDEAL technique (BMFV_IDEAL_ and BMFF_IDEAL_, respectively) in children with cerebral palsy (CP) and in typically developing children (Con). Values are means ± SE. *Group difference, *p* < 0.05.

There were no significant group differences between children with CP and typically developing children in BMFV_T1_ (6.1 cm^3^ ± 3.1 cm^3^ vs. 6.9 cm^3^ ± 1.9 cm^3^; *d* = 0.284, *p* = 0.439) or BMFV_IDEAL_ (6.1 cm^3^ ± 3.0 cm^3^ vs. 6.8 cm^3^ ± 1.9 cm^3^; *d* = 0.286, *p* = 0.438; Fig. 4A). Although BMFF_IDEAL_ was higher in children with CP than in typically developing children (82.1 ± 2.4 vs. 80.3 ± 2.0; *d* = 0.816, *p* = 0.034), there was no significant group difference in BMFF_T1_ (81.9 ± 3.2 vs. 80.8 ± 2.3; *d* = 0.409, *p* = 0.272; Fig. 4B).

## Discussion

The primary finding was that bone marrow fat volume estimates based on standard T1-weighted magnetic resonance images agreed extremely well with estimates based on fat and water images generated from the IDEAL technique in both typically developing children and in children with CP. However, bone marrow fat fraction estimates based on standard T1-weighted images were only moderately correlated with estimates based on fat and water images from the IDEAL technique. Moreover, the technique using standard T1-weighted images did not detect a higher bone marrow fat concentration in children CP compared to typically developing children, which was detected using fat and water images from the IDEAL technique. The finding suggests that the technique using standard T1-weighted images is less sensitive to variations in fat concentration in bone marrow. The source of errors for the BMFV_T1_ and BMFF_T1_ can probably be attributed to both partial volume effects and the use of a single threshold for bone marrow fat segmentation^29^.

The assessment of bone marrow fat by the IDEAL technique has been validated using MRS. Excellent agreement between bone marrow fat by the IDEAL technique and by the MRS technique has been observed using a phantom^30^ and human vertebra^31^. MRS is the most commonly used technique to quantify bone marrow fat in humans, especially at the vertebral site^32–35^. However, compared to MRS, the IDEAL technique has several advantages. One advantage is its shorter scan time^36^ because it does not require a long shimming time to make the magnetic field more homogeneous. This is particularly important when imaging clinical populations who may have a difficult time holding still over an extended period of time, such as children with spastic CP. In addition, compared to the most widely used single-voxel MRS method, the IDEAL method can provide much greater coverage because it is not limited by the size of the voxel. Due to these advantages, the IDEAL method may be a better choice than the MRS method when assessing bone marrow fat for clinical and research purposes, especially when assessing children.

To our knowledge, this is the first human study to assess the validity of bone marrow fat fraction estimates based on standard T1-weighted images. Previous studies have quantified bone marrow fat using T1-weighted magnetic resonance images^17,18,29^. Shen *et al*.^17^ found that pelvic bone marrow fat volume and total body bone marrow fat volume determined using T1-weighted magnetic resonance images similar to the images described in the present study were both inversely and significantly associated with total body BMD in Caucasian women. This observation supports the link between high bone marrow fat and low bone density. Similar associations between bone marrow fat volume from T1-weighted images and total body BMD was also observed in both younger and older adults^18^. Another study tested the validity of using T1-weighted magnetic resonance images to quantify bone marrow fat at the third lumbar vertebra and the femoral neck in post-menopausal women using the MRS and the DIXON methods for comparison^29^. Bone marrow fat volume from T1 images was strongly related to bone marrow fat fraction from the MRS method (*r* = 0.88, *p* < 0.001 for vertebra; femoral neck MRS measurement was not acquired) and from the DIXON method (*r* = 0.79, *p* < 0.001 for vertebra and *r* = 0.86, *p* < 0.001 for femoral neck). However, the relationship between bone marrow fat fraction from T1-weighted images and the other methods was not reported. Moreover, to date, no studies have examined the validity of using T1-weighted magnetic resonance images to quantify bone marrow fat in children.

The ability to assess bone marrow fat *in vivo* is of particular interest to many researchers because of its well-established connection with osteoporosis. Furthermore, bone marrow fat has been suggested to play a role in the pathogenesis of human metabolic risks. However, the mechanisms underlying the effect of bone marrow fat on metabolism are complicated and yet to be elucidated. An increase in bone marrow fat could potentially lead to a reduced number of available hematopoietic stem cells by simply taking over the available spaces, as well as by negatively influencing the microenvironment of these cells^37^. This is important because hematopoietic stem cells only utilize glucose (glycolysis) as energy resources^38^. Thus, those with elevated bone marrow fat may have a higher risk of developing type 2 diabetes, and bone marrow fat may serve as a surrogate marker for early signs of glucose dysregulation; although more studies are needed to confirm this notion.

The significant correlations found in the current study between BMFV_T1_ and BMFV_IDEAL_ in both typically developing children and children with CP indicate that bone marrow fat as estimated with T1-weighted magnetic resonance images can yield good volumetric measures in both groups. However, in the pediatric population, bone marrow may keep expanding simply due to growth and maturation. Therefore, bone marrow fat fraction compared to bone marrow fat volume may be a more appropriate measure of bone marrow fat infiltration in cross-sectional and longitudinal studies involving children^9,39^.

Unfortunately, the correlations between BMFF_T1_ and BMFF_IDEAL_ observed in typically developing children and in children with CP were only moderate. More importantly, the between group comparison of BMFF_T1_ was not significant and the effect size was only small to moderate (*d* = 0.409). On the other hand, BMFF_IDEAL_ was significantly higher in the children with CP than in the typically developing children and the effect size was large (*d* = 0.816). It is estimated that 58 participants per group would be needed to detect a significant between group difference in BMFF_T1_; whereas, only 15 participants per group were needed to detect a difference in BMFF_IDEAL_. Together, the correlational data and the group comparison data suggest that when compared to the IDEAL technique, the technique based on T1-weighted magnetic resonance images may be less sensitive to variations in the bone marrow fat fraction.

The limitations of the study must be considered. It is assumed that the bone marrow fat fraction estimates based on the fat and water images generated using the IDEAL technique provided more accurate estimates of bone marrow fat fraction than the technique based on standard T1-weighted images. Although the accuracy of any *in vivo* technique has limitations, bone marrow fat fraction estimates from fat and water images and the IDEAL technique have been validated against the MRS technique^30,31^, which is considered the most accurate *in vivo* method for assessing bone marrow fat fraction^30^. In addition, our intent was to capture the fat content within the entire bone marrow area, and due to the shape of bone marrow, such a goal is hard to achieve with the single-voxel MRS technique.

## Conclusion

A procedure using standard T1-weighted magnetic resonance images can produce estimates of bone marrow fat volume similar to estimates from fat and water images generated using the IDEAL technique in children. However, it is less sensitive to variation in the bone marrow fat fraction.
